# Assisted curation of regulatory interactions and growth conditions of OxyR in *E. coli* K-12

**DOI:** 10.1093/database/bau049

**Published:** 2014-06-04

**Authors:** Socorro Gama-Castro, Fabio Rinaldi, Alejandra López-Fuentes, Yalbi Itzel Balderas-Martínez, Simon Clematide, Tilia Renate Ellendorff, Alberto Santos-Zavaleta, Hernani Marques-Madeira, Julio Collado-Vides

**Affiliations:** ^1^Programa de Genómica Computacional, Centro de Ciencias Genómicas, Universidad Nacional Autónoma de México, A.P. 565-A, Cuernavaca, Morelos 62100 and ^2^Institute of Computational Linguistics, University of Zurich, Binzmuhlestrasse 14, Zurich 8050, Switzerland

## Abstract

Given the current explosion of data within original publications generated in the field of genomics, a recognized bottleneck is the transfer of such knowledge into comprehensive databases. We have for years organized knowledge on transcriptional regulation reported in the original literature of *Escherichia coli* K-12 into RegulonDB (http://regulondb.ccg.unam.mx), our database that is currently supported by >5000 papers. Here, we report a first step towards the automatic biocuration of growth conditions in this corpus. Using the OntoGene text-mining system (http://www.ontogene.org), we extracted and manually validated regulatory interactions and growth conditions in a new approach based on filters that enable the curator to select informative sentences from preprocessed full papers. Based on a set of 48 papers dealing with oxidative stress by OxyR, we were able to retrieve 100% of the OxyR regulatory interactions present in RegulonDB, including the transcription factors and their effect on target genes. Our strategy was designed to extract, as we did, their growth conditions. This result provides a proof of concept for a more direct and efficient curation process, and enables us to define the strategy of the subsequent steps to be implemented for a semi-automatic curation of original literature dealing with regulation of gene expression in bacteria. This project will enhance the efficiency and quality of the curation of knowledge present in the literature of gene regulation, and contribute to a significant increase in the encoding of the regulatory network of *E. coli*.

**RegulonDB Database URL:** http://regulondb.ccg.unam.mx

**OntoGene URL:** http://www.ontogene.org

## Introduction

Genomics is facing the challenge to accelerate the pace of facilitating the access to large amounts of knowledge in forms adequate for human understanding. Biocuration is an activity at the core of transferring knowledge contained within original scientific literature to organized databases. RegulonDB is one of the primary databases initially published >15 years ago containing high-quality manually curated knowledge on, the regulation of transcription initiation in *Escherichia coli* K-12, the microbial organism with the largest corpus of publications (1, 2).

RegulonDB contains objects such as genes, promoters, transcription factors (TFs), transcription factor binding sites (TFBSs), terminators and operons. It also contains relationships of regulation among TFs and genes, promoters and operons that describe how a TF regulates in a positive, negative or dual way the expression of a gene at the level of transcription initiation. These types of relationships are called ‘regulatory interactions’ (RIs), which can be consider a tuple of the TF, effect, and target gene or transcription unit within specified experimental conditions (ECs). The precise content of an RI depending on the knowledge available may include only the target gene, its induction or repression, the TF and the condition under which this change of expression happens, or it may specify the regulated promoter and the set of TFBSs involved in the regulatory mechanism.

Keeping this information up to date has involved for years the work of around three full-time curators. It is true that their work has involved not only reading and extracting the knowledge from papers, but also participating in discussions to enrich our model of curation, increasing the set of properties that we extract from the literature, ways of better associating references, the incorporation of gene ontologies, the adaptations that novel technologies impose and the design of navigation, all essential activities in our electronic editing of gene regulation. In spite of the size of our curation team, we can barely cope with maintaining up to date the curation of transcriptional regulation for only one bacterium, *E. coli* K-12. We are, therefore, strongly motivated to modify our manual curation strategies, taking advantage of the benefits of natural language processing implementations that could enhance our performance to expand and enrich the quality and quantity of curated knowledge. In this work, we report our initial results from use of such technologies guided by our aim of encoding growth conditions.

Gene regulation enables the cell to adapt to changes in the environment. A fundamental piece of information that we have gathered from only a small number of papers is that of the conditions under which the regulatory mechanisms have been studied, more precisely the experimental conditions (ECs) and their corresponding control conditions (CCs) used in the experiments to identify RIs. For simplicity, we refer to growth conditions (GCs) here, the pair of ECs and CCs.

The process of curation of GCs would require keeping track of the name of the GC, the control of the experiment, the growth media used, the temperature, the pH, the type of effect (induction or repression) on the regulated genes provoked by the transition from CC to EC, and the TF and sigma factor involved (when known) in such regulatory mechanisms. Thus, the biocuration challenge we face is to extract this type of relevant information from the large corpus of around 5000 papers currently curated by RegulonDB. This set of articles supports the knowledge of mechanisms present in RegulonDB with experiments performed since the 1980s, or even 1970s, to date. Doing this work manually would require a considerable amount of time. This specific challenge motivated us to initiate the collaboration between RegulonDB scientists and the OntoGene group, leveraging on their expertise in text-mining tools. This collaboration allowed our RegulonDB team to use the text-mining tools developed by the OntoGene group and their assisted curation interface (ODIN) to simplify and accelerate the curation process.

OntoGene/ODIN provides a flexible, customizable environment for document-centric curation approaches. OntoGene/ODIN has been previously described in several publications (3–5). The OntoGene team at the University of Zurich, working in collaboration with RegulonDB curators, adapted and expanded ODIN for the specific needs of this project.

We here report a proof of concept project that addresses the extraction of RIs and GCs from the set of 48 papers cited by RegulonDB supporting the regulation of gene expression by OxyR, the transcriptional regulator for the expression of antioxidant genes in response to oxidative stress and, in particular, to elevated levels of hydrogen peroxide (6, 7). OntoGene/ODIN has been customized for the curation of gene regulation in *E. coli*. The set of selected papers were processed and made available to curators in the OntoGene/ODIN interface. The curators focused on RIs and their corresponding GCs, extracting informative sentences, as opposed to reading the complete papers. Our manual-assisted curation shows highly positive results as evaluated with interactions present in RegulonDB supported by these 48 papers on one hand, as well as with the specific and less detailed GCs validated by curators.

## Materials and methods

The OntoGene text-mining system contains modules for entity recognition and relation extraction, based on rule-based approaches (e.g. lexical lookup with variants) as well as machine-learning approaches (e.g. maximum entropy techniques). For the initial annotation of domain entities (genes, TFs, GCs, etc) in the RegulonDB papers, we used only a simple lexical lookup approach for named entity extraction.

We used the complete list of genes of *E. coli* Version NC_000913.2 GI:49175990 from GenBank and contained in RegulonDB (http://regulondb.ccg.unam.mx/menu/download/datasets/files/GeneProductSet.txt) for building dictionaries to be used by OntoGene for the automated annotation. Additionally, RegulonDB provides words that indicate the type of effect caused under a given GC (‘activation’, ‘repression’ and a complete list of their synonyms), which were also added to the terminological base of OntoGene. We worked with 48 articles that were identified in RegulonDB version 8.5 given the references to all objects, RIs, operons, promoters and terminators of genes subject to OxyR regulation. Papers were extracted from PubMed in different formats (PDF, XML) and processed using the PDFlib Text Extraction Toolkit for text manipulation (http://www.pdflib.com/?id=12). These articles were automatically annotated by using the OntoGene pipeline and the terminology provided by RegulonDB, which includes types such as gene, effect, TFs, etc.

The set of sentences, individually enumerated, contained within those papers was then viewed through ODIN (OntoGene Document Inspector), a flexible browser-based client application that interfaces with the OntoGene server. The curator then used the facilities provided by ODIN to visualize selected annotations, and, if necessary, modify them.

**Figure 1.**
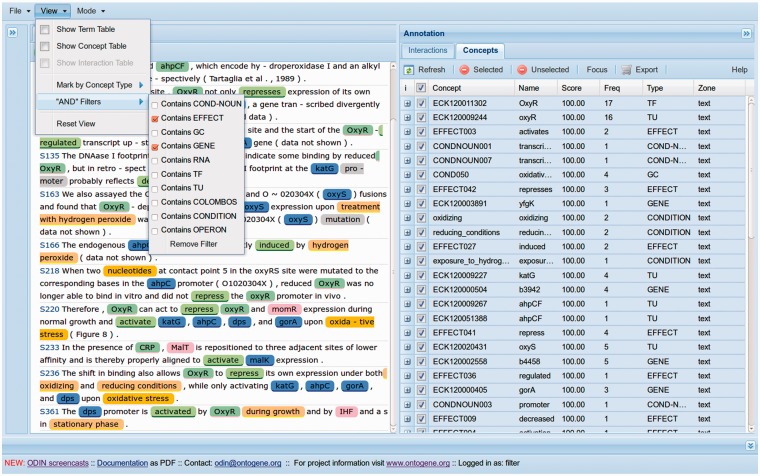
The display of ODIN adapted for curation in RegulonDB.

The OntoGene team implemented a new capability within ODIN called ‘sentence filters’, that allows users to specify a simple logical condition that functions as a filter, i.e. only sentences satisfying the specified condition are listed (Figure 1). The curator used these filters to select and visualize, in those 48 articles, only those sentences containing the name of a target gene and a word for the type of effect. Our hypothesis was that the GCs would be part of sentences containing the target gene and the effect of the TF. Once these sentences were extracted, a series of analyses were performed as shown in the following sections.

## Results and discussion

From the 48 papers related to OxyR, we found that 43 papers contributed with at least one sentence after applying the filter in ODIN that requires a target gene and the effect; the other five papers did not contribute any further to this analysis. We verified that these five papers do not contain information about any RI or GC, although they have information about promoters and other elements related to the content of RegulonDB.

The 48 papers contain 12 037 sentences, and we read only 1375 sentences that were the result of applying the filter. That is, we read only 11.4% of the sentences in these papers. We obtained information on GCs and RIs from 550 of the 1375 sentences. The remaining 825 sentences contain information about other types of regulation, such as regulation by small RNAs and regulation of activity of proteins. This is valuable information that we can process to expand our curation beyond our current focus on regulation of transcription initiation.

### Normalized structures derived from sentences.

Our first observation was the rich variety of linguistic expressions used to describe the same content. Certainly, authors use different words to express the same idea. Thus, we considered it a necessity to create a common representation to normalize the information irrespective of the original wording of the sentences in a paper. Based on the analyses of the complete set of informative sentences focused on extracting information on GCs, we mapped all informative sentences into three different normalized structures:

- ‘TF [effect] gene [GC]’
- ‘TF [effect] gene’
- ‘[effect] gene [GC]’

The first structure is the most complete, it has information about the TF that positively or negatively (effect) regulates the target gene(s) and the GC(s) used. The other two structures are less informative. The second structure is similar to the first except that it does not contain the GC, whereas the third one has no TF included. The last two structures are also worth curating as not all regulatory mechanisms are equally well-known, and this partial information will most likely be, later on, complemented with other sentences and new papers.

When these structures are mapped to specific sentences, they look, for example, as follows: ‘MntR [+] *mntH* [manganese]’, which reads as follows: the MntR TF positively regulates the expression of the *mntH* gene in the presence of manganese. Table 1 illustrates four different instances of this semantic structure in the same paper (8).

**Table 1.** The same sematic structure obtained from several sentences

Sentence	Semantic structures
We also discovered that the ***mntP*** gene (formerly *yebN*), encoding a putative efflux pump, is **upregulated** by **manganese** through **MntR**.	MntR [+] *mntP* [manganese]
Taken together, the observations that ***mntP*** is **upregulated** by **manganese** through **MntR**, while its deletion causes dramatic sensitivity to manganese and increased intracellular manganese levels indicate that MntP functions as an efflux pump.	MntR [+] *mntP* [manganese]
We additionally found that expression of the ***mntP*** gene is **upregulated** by **manganese** through **MntR**.	MntR [+] *mntP* [manganese]
We demonstrated **MntR**-dependent **upregulation** of ***mntP*** upon exposure to **manganese** (Figure 8), indicating that in addition to repressing gene expression, MntR can positively regulate transcription.	MntR [+] *mntP* [manganese]

The words in bold are those extracted in the corresponding normalized semantic structures.

A total of 898 instantiated structures were derived from the 43 papers. It should be noted that the relationship between a sentence of a paper and its corresponding structure is not always going to be 1:1. One sentence may require two or more structures to represent all its information. For example, we could extract the structures shown in Table 2 from the following sentence: ‘The two binding modes probably allow OxyR to repress the oxyR and mom promoters during normal growth, while activating katG, ahpCF, dps, gorA, and oxyS only upon oxidative stress’ (9).

**Table 2.** Several semantic structures obtained from a unique sentence

Sentence	Semantic structure
The two binding modes probably allow **OxyR** to **repress** the **oxyR** and mom promoters during normal growth, while activating katG, ahpCF, dps, gorA and oxyS only upon oxidative stress.	OxyR [−] oxyR
The two binding modes probably allow **OxyR** to repress the oxyR and mom promoters during normal growth, while **activating katG**, ahpCF, dps, gorA and oxyS only upon **oxidative stress**.	OxyR [+] katG [oxidative stress]
The two binding modes probably allow **OxyR** to repress the oxyR and mom promoters during normal growth, while **activating** katG, **ahpCF**, dps, gorA and oxyS only upon **oxidative stress**.	OxyR [+] ahpCF [oxidative stress]
The two binding modes probably allow **OxyR** to repress the oxyR and mom promoters during normal growth, while **activating** katG, ahpCF, **dps**, gorA and oxyS only upon **oxidative stress**.	OxyR [+] dps [oxidative stress]
The two binding modes probably allow **OxyR** to repress the oxyR and mom promoters during normal growth, while **activating** katG, ahpCF, dps, **gorA** and oxyS only upon **oxidative stress**.	OxyR [+] gorA [oxidative stress]
The two binding modes probably allow **OxyR** to repress the oxyR and mom promoters during normal growth, while **activating** katG, ahpCF, dps, gorA and **oxyS** only upon **oxidative stress**.	OxyR [+] oxyS [oxidative stress]

The words in bold are those extracted in the corresponding normalized semantic structures.

Note that our mapping rules associate a specific sentence with the most complete possible structure. However, as shown, a single sentence may instantiate different structures with different regulatory content (interactions). In this example, the following structures were not included: (i) the (OxyR [−] mom) because the mom gene is absent in *E. coli*; (ii) (OxyR [−] *oxyR* normal growth) because ‘normal growth’ is not informative.

As shown below, these normalized structures simplify the interpretation of the data and enable the comparison and counting of sentences with the same meaning, irrespective of the flexibility in expression that ocurs in natural language.

### Homogenization and structuring of terminology

A similar effort of normalization at the level of individual words is required for effect, GC, gene and TF. To express that a gene is activated, terms such as ‘activated transcription’, ‘initiated transcription’ and ‘increased transcription’ are used among several others. Similarly, GCs can be expressed in several ways and at different levels of detail. For example, ‘oxidative stress' and ‘exponential phase' might appear in different sentences with different words, such as:

- oxidative stress, oxidizing conditions, oxidants, oxidant
- during growth, exponentially growing, exponential growth, exponential phase, exponentially growing cells, logarithmic phase, log phase

We identified all the synonymous terms of GCs, and selected one as the reference, and then we replaced the synonyms with the reference in our semantic structures. In the cases shown above, we selected ‘oxidative stress’ for the first set of terms, and ‘exponential phase’ for the second set of terms, as these were the most frequently used in the corpus.

A separate issue is the benefits of using the hierarchical structure in the ontology of the different levels of detail used to describe GCs. Efforts such as those of COLOMBOS and PortEco, dealing with repositories of microarray experimental data, use either use previous ontologies or develop their own (10, 11). Thanks to an initial collaboration with the COLOMBOS team, we are using their descriptions as an alternative dictionary of conditions within ODIN (See Figure 1).

For example, ‘oxidative stress’, can be studied using names of various chemical compounds such as hydrogen peroxide and paraquat, when they are in the growth medium. Therefore, when a gene is analyzed and its expression is affected under treatment with hydrogen peroxide, it is relatively common to find expressions such as ‘the gene is activated by oxidative stress’ and ‘the gene is activated by hydrogen peroxide’. Among the search terms, oxidative stress and hydrogen peroxide are actually two ways to describe the same experiment in that paper, with the only difference being that the former is expressed using a more general term, and the latter using a more specific term.

Synonyms are also used for TFs and gene names. We used the same mapping of synonyms to the main name of the gene or TF based on the names of objects commonly used in RegulonDB and in EcoCyc (12).

Having solved the problems of the variety of sentences with the same meaning and synonyms for elements involved in RIs, we were able to initiate the analysis of the content of all the informative sentences extracted from the corpus of OxyR literature.

### OxyR RIs and their GCs

The purpose of this work was to validate the results derived from reading only the sentences selected automatically within ODIN, instead of reading the complete paper, as has been done for years in curating RegulonDB. We examined the selected sentences from the 43 articles and obtained the structures derived from each one of them. They were collected and compared in order to analyze what type of information they expressed.

We initially obtained the list of the 21 RIs of OxyR from RegulonDB (Table 3), and we compared them with the information in the semantic structures from this curation experiment when it contained the token ‘OxyR’, e.g., ‘OxyR [+] *oxyS*’ or ‘OxyR [+] *trxC* [hydrogen peroxide treatment]’. We realized that through assisted curation and review of only the filtered informative sentences, we could identify all 21 RIs of OxyR contained in RegulonDB.

**Table 3.** OxyR RIs and their GCs

Interaction number	GENE/operon target	EFFECT	GC
1	fhuF	Repressor	Hydrogen peroxide treatment
2	flu	‘Activator/repressor’	Hydrogen peroxide treatment
3	gor	Activator	Hydrogen peroxide treatment
4	grxA	Activator	Hydrogen peroxide treatment
5	katG	Activator	Hydrogen peroxide treatment
6	mntH	Activator	Hydrogen peroxide treatment
7	oxyS	Activator/repressor	Hydrogen peroxide treatment
8	sufABCDSE	Activator	Hydrogen peroxide treatment
9	trxC	Activator	Hydrogen peroxide treatment
10	uof-fur	Activator	Hydrogen peroxide treatment
11	ychF	Repressor	Hydrogen peroxide treatment
12	ahpCF	Activator	Hydrogen peroxide treatment, ascorbate treatment
13	dps	Activator	Hydrogen peroxide treatment, exponential phase, stationary phase
14	gntP	Repressor	Not found
15	hcp-hcr	Activator	Not found
16	uxuAB	Repressor	Not found
17	ybjC-nfsA-rimK-ybjN	Repressor	Not found
18	yhjA	Activator	Not found
19	dsbG	Activator	Oxidative stress
20	hemH	Activator	Oxidative stress
21	oxyR	Repressor	Oxidative stress, reducing conditions

List of all RIs for OxyR extracted from the corpus. The RIs that show a dual effect ‘activator/repressor’ are discussed in the text. For some RIs, no GC was found.

In addition, we could correctly identify the effect of 19 of the 21 RIs, while in the two remaining RIs (OxyR [−] flu) and (OxyR [+] *oxyS*), we could identify in the corpus some sentences describing positive effects and other describing negative effects for these two interactions, see Table 3.

We found sentences describing a positive and a negative effect of OxyR on the *flu* gene. However, 65 sentences described a negative effect, and only one described a positive effect, and in three cases the effect was described as ‘not repression’. Therefore, the effect is very likely to be negative, exactly as it is reported in RegulonDB. We assumed that the sentences mentioning a positive effect referred to a mutant. A review of the articles confirmed that these sentences were describing mutants not capable of suppressing the *flu* gene. This illustrates the need for context by including additional sentences for a precise curation, especially because mutants are frequently used to provide evidence for RIs.

The other instance in which (OxyR [+] *flu*) occurred was in a paper suggesting that besides the known negative effect, there is an indirect positive role of OxyR on *flu*. This apparent error was corrected by the curator. In fact, indirect effects are not included in RegulonDB.

Another case of inconsistency is that of the OxyR [+] *oxyS* interaction*.* In a sentence it was mentioned that *oxyS* is repressed by OxyR; however, the reference associated with this sentence clearly stated that *oxyS* is activated, not repressed. Thus, this is not an error of our system, but a consequence of an erroneous citation. It is worth noting that 18 sentences mentioned that OxyR activate *oxyS*, against only one indicating a repression. These few inconsistencies, here limited to two interactions out of 21, show the necessity of the attentive work required of a curator.

In addition to the RIs of OxyR already present in RegulonDB, we also extracted 14 RIs of OxyR that are not present in RegulonDB; those in which OxyR regulates the genes *yaaA*, *yaeH*, *yaiA*, *ybjC*, *ybjM*, *ydcH*, *ydeN*, *yfiA*, *ygaQ*, *ygaR*, *yhjA*, *yljA*, *ytfK* and *hemF-rcsC*. The first 13 interactions were reported in a single article describing an experiment of transcriptome analysis. Given our more strict curation criteria, such cases with a weak evidence are incorporated into RegulonDB only if they contain additional evidence such as the identification of the DNA-binding site for the TF (13). The last of the RIs (OxyR activates the expression of genes for the unit of transcription hemF-rcsC) was found in an article that cited a work from 1994, which had escaped our curation. This showed that the use of assisted curation can help to identify gaps in the conventional process of manual curation.

The main goal of this current work is to extract GCs missing in RegulonDB. In this work, we identified the GCs for 16 of the 21 RIs of OxyR. The predominant condition is ‘hydrogen peroxide treatment’ (Table 3, fourth column), indicating that our results are correct, as the OxyR protein is normally active under the condition of ‘oxidative stress’ (6, 7). It is also active under conditions of reduction, in which case it represses itself (14), which is also shown in our results (Table 3, interaction 21). Identification of a predominant condition suggesting that the capability to inspect a set of articles about a specific TF, instead inspecting of each single article in isolation, might be a more reliable way to validate the results for GCs because most TFs are active or inactive under few specific conditions.

These initial results already show the advantages of using an assisted curation environment. By assisted curation we mean a combination of text-mining approaches and the work of an expert curator. In terms of RIs, our assisted curation recuperated 100% of all OxyR RIs with no false positives, and 76% of their corresponding GCs and no false positives. We assume that besides the 1375 phrases automatically extracted, there are many that may refer to RIs of a variety of TFs, including OxyR. We know there are sentences describing in a different linguistic context the same interactions of OxyR. However, missing them did not affect our final result of obtaining all RIs and most of the GCs. This means that there may be other linguistic structures that could work equally well to those we designed.

### Additional RIs and their GCs

In this same corpus of papers, in addition to the OxyR RIs, we identified 29 RIs for a variety of TFs, with their corresponding GCs. These RIs involved the TFs AraC, ArcA, CRP, Fis, FNR, Fur, IHF, IscR, MntR, NarL, NarP, SigmaS and SoxS (Table 4). We also found 75 RIs for which we could not identify the GCs used in their identification (for the TFs ArcA, CaiF, CRP, CysB, Fis, FNR, Hns, IlvY, LexA, Lrp, LysR, MalT, MarA, MetR, NarL, NarP, PhoB, Sigma70, SigmaS, SoxR and SoxS) (data not shown). Additionally, we identified 67 associations between GCs and target genes where the regulator executing the regulatory effect was missing in the literature. The incomplete RI tuples are shown in Table 5. Note that in these cases we used ‘induction’ and ‘inhibition’ for referring to inducible and repressible systems, as used in the literature of microbial physiology when the precise activator or repressor has not yet been identified.

**Table 4.** RIs and their GCs of several TFs

TF name	GENE/operon name	EFFECT	GC
AraC	ara	Repressor	Absence of arabinose
ArcA	cydAB	Activator	Anaerobiosis
ArcA	gltA	Repressor	Anaerobiosis
ArcA	sdhCDAB	Repressor	Anaerobiosis, anoxic transition
ArcA	sodA	Repressor	Anaerobiosis
CRP	oxyR	Activator	Exponential phase
Fis	acs	Repressor	Exponential phase
FNR	yfgF	Activator	Anaerobiosis
FNR	yhjA	Activator	Anaerobiosis
Fur	fhuF	repressor	Iron-rich conditions + absence of oxidative stress
Fur	mntH	Repressor	CO_2_ treatment, iron treatment
Fur	sufABCDSE	Repressor	Iron treatment
IHF	acs-yjcH-actP	Repressor	Stationary phase
IHF	sufABCDSE	Activator	Oxidative stress
IHF + SigmaS	dps	Activator	Stationary phase
IscR	iscRSUA	Repressor	Anaerobiosis, reactive oxygen species
MntR	dps	Repressor	Stationary phase
MntR	mntH	Repressor	Manganese treatment, iron treatment, metal treatment
MntR	mntP	Repressor	Manganese treatment
MntR	mntS	Repressor	Manganese treatment
NarL	hcp-hcr	Activator	Nitrate treatment, Nitrite treatment
NarP	hcp-hcr	Activator	Nitrate treatment, Nitrite treatment
SigmaS	aidB	Activator	Oxygen-limiting conditions
SigmaS	ansP	Activator	Onset of stationary phase
SigmaS	artIPQM	Activator	Onset of stationary phase
SigmaS	ilvD	Activator	Onset of stationary phase
SigmaS	tnaA	Activator	Onset of stationary phase
SoxS	mutM	Activator	Superoxide generators treatment
SoxS	ybjC-nfsA-rimK-ybjN	Activator	Paraquat treatment

These are interactions obtained from the same corpus of papers.

**Table 5.** GCs and their effect on target genes

GC	EFFECT	GENE/operon name
2,2′-dipyridyl treatment	Induction	mntH
2,2′-dipyridyl treatment	Inhibition	mntS
2,2′-dipyridyl treatment	Induction	isc
2,2′-dipyridyl treatment	Induction	suf
Aerobiosis	Induction	katG
Anaerobiosis	Induction	arcA
Anaerobiosis	Induction	hcp
Anaerobiosis	Inhibition	lctPRD
Anaerobiosis + nitrate treatment	Induction	hcp
Anaerobiosis + nitrite treatment	Induction	hcp
Carbon starvation	Induction	csiD
Δfur mutant	Induction	ryhB
ΔoxyR mutant	Induction	sufA
EDTA treatment	Induction	mntH
Exponential growth	Induction	bolA
Fructuronate treatment	Induction	uxuAB
Gluconate treatment	Inhibition	gntP
Glucose treatment	Inhibition	gntP
Glucose treatment	Inhibition	oxyR
Glucose treatment	Inhibition	uxuAB
Glucuronate treatment	Induction	uxuAB
Hydrogen peroxide treatment	Induction	fpr
Hydrogen peroxide treatment	Induction	iscRSUA
Hydrogen peroxide treatment	Inhibition	rplB
Hydrogen peroxide treatment	Induction	sodA
Hydrogen peroxide treatment	Induction	soxS
Hydrogen peroxide treatment	Induction	yaeH
Hydrogen peroxide treatment	Induction	ydcH
Hydrogen peroxide treatment	Induction	ydeN
Hydrogen peroxide treatment	Inhibition	yfdI
Hydrogen peroxide treatment	Induction	ygaQ
Hydrogen peroxide treatment	Induction	ytfK
Hydrogen peroxide treatment	Inhibition	fldA
Hydrogen peroxide treatment + Δfur mutant	Induction	sufA
Iron starvation	Induction	mntH
Iron starvation	Induction	isc
L-ascorbate + glutamine treatment	Induction	yiaK
L-ascorbate + proline treatment	Induction	yiaK
L-ascorbate + threonine treatment	Induction	yiaK
L-ascorbate treatment	Induction	ulaA
L-ascorbate treatment	Induction	ulaG
L-ascorbate treatment	Induction	yiaK
L-ascorbate treatment + early exponential phase	Regulation	ahpC
Mannonic amide treatment	Induction	uxuAB
Menadione treatment	Induction	ahpC
Menadione treatment	Induction	ryhB
Menadione treatment	Induction	sufA
Menadione treatment + DoxyR	Induction	sufA
Menadione treatment + DsoxRS	Induction	sufA
Menadione treatment + DsoxS	Induction	sufA
Mncl2 treatment	Induction	sodA
Nitrosative stress	Induction	hcp-hcr
Nitrosative stress	Induction	hmp
Nitrosative stress	Induction	yeaR-yoaG
Nitrosative stress	Induction	ytfE
Paraquat treatment	Induction	fldA-fur
Paraquat treatment	Induction	oxyS
Paraquat treatment	Induction	soxS
Paraquat treatment	Induction	sufA
Plumbagin treatment	Induction	sufA
PMS treatment	Induction	ahpC
PMS treatment	Induction	katG
PMS treatment	Induction	ryhB
PMS treatment + Dfur mutant	Induction	sufA
Stationary phase	Induction	katE
Stationary phase	Inhibition	oxyR
Superoxide treatment	No effect	mntH

As discussed in the text, ‘induction’ and ‘inhibition’ are used here because there is no knowledge about the mechanism or TF involved in these interactions.

Within these interactions, we found some genes whose expression is induced by hydrogen peroxide; therefore, such genes can be considered as candidates for OxyR regulation.

Within this whole set of 105 RIs (29 + 75), we found 94 in RegulonDB and only 10 were missing in the database. One of them is an indirect effect, four are proposed based on microarray experiments, and four have not been curated in the database.

#### Excluded interactions

Some interactions among target genes, TFs and GCs were not considered in these results for several reasons, essentially because of descriptions that we do not consider to be sufficiently informative. In some cases there is a lack of specificity in the effect, for instance when words like ‘regulated’, ‘not repressed’ or ‘not induced’ are used. We decided to ignore such cases and select only those sentences that mentioned whether the gene is activated or repressed. However, in some cases, the sentences containing only the term ‘regulated’ were useful to validate other sentences with the same gene or TF when the directionality of the effect (activated or repressed) was clearly stated. In some cases, we also considered sentences containing the assertion that ‘no effect’ was observed because we believe that it is quite important to also encode negative results.

Another case of excluded interactions is when the term for GC is too ambiguous, such as ‘the X gene is regulated by pH’. In addition to missing directionality for the effect, the GC (pH) in this case is underspecified.

Also, all the negative SigmaS interactions were excluded. Sigma factors are subunits of RNA polymerase and allow the recognition of the promoter to initiate transcription. Thus, the negative effect of sigma factors has to be in principle an indirect effect, and we do not systematically gather indirect effects in our curation routine. Finally, cases where the operon name was not clearly identified were also excluded (See the section on future improvements, below).

### Identification of the reference for the OxyR interactions

To include an RI in RegulonDB, we need to identify the original paper that provides the evidence for the RI between gene, TF and condition. In this section, we describe our initial work in this direction, with the aim of extracting the reference as part of the curation process within ODIN.

We have thought of several strategies to identify the original paper reporting a piece of knowledge. For example, we can assume that informative sentences with a reference are citing previous work (See ‘Reference’ in Table 6). Such cited paper could be the original paper.

**Table 6.** The (OxyR + katG) sentences in the corpus

Sentence_ID	Normalized structures	Information within the sentence	Paper
S23	OxyR [+] katG		PMID:10419964 (15)
S17	OxyR [+] katG		PMID:12644490 (16)
S165	OxyR [+] katG	Reference (PMID:2693740)	PMID:12644490 (16)
S116	OxyR [+] katG		PMID:15009899 (17)
S38	OxyR [+] katG	Reference (PMID:2693740)	PMID:1730735 (18)
S208	OxyR [+] katG	Reference	PMID:1730735 (18)
S147	OxyR [+] katG	Reference (PMID:8087856)	PMID:17464064 (19)
S157	OxyR [+] katG		PMID:17464064 (19)
S42	OxyR [?] katG		PMID:2693740 (20)
S140	OxyR [+] katG		PMID:2693740 (20)
S180	OxyR [+] katG	Evidence	PMID:2693740 (20)
S99	OxyR [?] katG		PMID:8990289 (21)
S141	OxyR [?] katG		PMID:8990289 (21)
S106	OxyR [+] katG		PMID:9324269 (22)
S27	OxyR [+] katG	Reference	PMID:11443091 (23)
S25	OxyR [+] katG	Reference	PMID:11443092 (24)
S94	OxyR [?] katG	Evidence	PMID:22539721 (25)
S118	OxyR [+] katG	Figure/Table	PMID:22539721 (25)
S20	H202 [+] katG	Reference	PMID:3045098 (26)
S20	OxyR [+] katG	Reference	PMID:3045098 (26)
S34	OxyR [?] katG	Reference	PMID:7868602 (27)
S15	OxyR [?] katG		PMID:7984106 (28)
S91	OxyR [?] katG	Reference linked	PMID:7984106 (28)
S135	OxyR [?] katG	Reference/evidence linked	PMID:8087856 (29)

The first column contains the ID of each sentence within the paper with PMID shown in the last column followed by their citation number in this paper. The third column indicates whether the sentence includes an evidence, a reference, an image or a table.

On the other hand, sentences which clearly state that the RI has been derived from a specific experiment, and also sentences containing references to figures or tables within the same paper, are likely to be describing experimental results obtained by the authors (See ‘Evidence’, and ‘Figure/Table’ in Table 6).

Another strategy that could be adopted to identify the reference for a RI is the frequency with which it is mentioned within a single paper. For example, if the same RI occurs more than three times in a paper, it is highly likely that interaction is supported by the experiments described in that article. However, it is not possible to draw the opposite conclusion, i.e. the fact that a result is mentioned only once does not imply that such a result is not an experimental result reported in that paper.

We tested these strategies with the case of *katG*. We identified assertions saying that the *katG* gene is positively regulated by OxyR in 24 sentences of 15 papers (Table 6), with three assertions in a single paper (PMID:2693740; sentences S42, S140 and S180). In fact, this last reference is correct (as verified in RegulonDB), and it contains assertions that mention the experimental evidence or method name used to verify it experimentally. The other two papers that contain experimental evidence within the sentence (PMID:22539721 and PMID:8087856), are also papers where the OxyR–katG interaction was experimentally validated.

Of the same 24 sentences, 11 have at least one reference. We checked such cited references, and three of them cite two papers with sentences containing the experimental evidence. As shown in Table 6, sentences S165 from paper PMID:12644490 and S38 from paper PMID:1730735 both cite the paper PMID:2693740, which contains sentence S180, which provides the evidence. Also, the reference cited in sentence S147 from the paper PMID: 17464064 is precisely the paper with PMID:8087856 that contains sentence S135, which provides evidence. The rest of references are citing other publications not included in our corpus, some of them are books or reviews.

These are positive preliminary results that remain to be tested when performing similar analyses with other sets of papers of different TFs.

## Future improvements

### Some yet-to-be-solved problems

#### Short gene and operon names

We noticed errors in the automated annotation of some gene names. In particular, short words (typically four letters or less) might also happen to be gene names. For example, ‘fold’ is frequently used to express the level of expression of a gene, rather than to refer to the gene of the same name. Such errors were manually corrected. A partial solution to this problem would be the use of capitalization rules for genes and proteins, even if this strategy would not work with three-letter gene names. However, there can be cases where the authors do not follow the nomenclature conventions, which would be missed if we enforced strict capitalization rules in OntoGene/ODIN.

In some cases, it is not possible to identify the names of the genes affected by the RI because often a shortened form of the name of the operon is used, which creates ambiguity (several operons share the same shortened form). For this reason, it is necessary to create a list of these shortened names and add it to ODIN’s annotation database.

We record the TFs with their proper names, such as ArcA, SoxR and OxyR. However, in some cases such TFs form part of a system, such as the system of the components in the case of ArcA. Several articles mention that a given gene is regulated by the ArcAB system. These cases cannot be currently identified with ODIN. However, adding the name of the system might solve the problem, as we know in this case that regulation is performed by ArcA because ArcB functions as a sensor and not as a DNA-binding transcriptional regulator. There are various similar cases, so we should identify the names that are used for such systems, create a list and store it in ODIN database so that they can be identified automatically. Additionally, we should create a way to automatically map the name of the system to the corresponding TF.

#### Limitations of single-sentence curation

Another problem to solve is the lack of clarity when a mutant is mentioned in a sentence without its description in the same sentence because the sentence-based curation approach hides the information needed for complete understanding (being it contained in a nonselected sentence). A similar problem is caused by anaphoric expressions such as the phrase ‘this gene’, where the actual name of the gene is mentioned in a previous sentence. We will modify ODIN to enable the user to inspect the previous and subsequent sentences when needed, to offer a larger context for curation.

A long-term goal is to use the system for a more specific, accurate and efficient curation. In the process of the curation experiment discussed here, we realized that it would be useful to distinguish interrogative or hypothetical sentences from affirmative ones because only the latter provide reliable data for curation.

We will continue and expand the activities described in this paper within the scope of a planned collaborative project with curators of the RegulonDB group and the OntoGene team. We will create new strategies to use ODIN’s filters to identify other types of information, for example, the conformation in which the TF is active, as we were able to identify several such cases during the work described in this article. For example, we often found the words ‘reduced’ and ‘oxidized’, which could be automatically marked as effectors, and generate the OxyR-reduced and OxyR-oxidized conformations. We could also direct the search towards the regulation of the activation and deactivation of TFs, which will require specific strategies.

### Towards the automation of the process

Overall, our results show that ODIN is a very useful instrument to help in what we call assisted-manual curation of RegulonDB.

Our future goal is to advance towards automating several steps of the curation process. Some observations made during this study will help to generate improved versions of the tools and terminological resources for this purpose. For example, because all TFs are also genes, they belong to two dictionaries in ODIN. However, if we want to curate only GC-related sentences, it would be better not to include the TFs in the list of genes used as a filter because the terms related to an effect found in a sentence with a GC are also found in sentences that contain only information on RIs. It is true that we may need to implement a different strategy so that we do not lose RIs of autoregulation. We also found GC-related data that regulate the activity of the TFs, and their mechanisms, even if not necessarily at the level of transcription (data not shown). This is also useful information for RegulonDB.

In this work, all informative sentences were read by a human expert curator, who had to perform additional tasks to generate the final data to feed RegulonDB. An important observation is that an automated curation process should not be based on single articles, but instead on sets of related articles, for example, all those discussing a specific TF, as here illustrated here. It would then be possible to aggregate information across different articles, for example, taking into account the frequency of a given assertion, to increase the reliability of the results. The confidence of such results would increase with the number of articles. The case of the *flu* gene regulated by the OxyR TF, we mentioned above is a good example, with ‘negative regulation’ mentioned 65 times, whereas mention of ‘positive regulation’ occurred only once.

In addition to the frequency of sentences representing the same information, we can also use sentences with partial information, but that are complementary among them. For example, if only a sentence mentions that MntR positively regulates *mntP* in the presence of manganese (‘MntR [−] *mntS* [manganese]’), but there are many other sentences that contain a partial presentation of the same information (‘[MntR [−] *mntS*] or [−]*mntS* [manganese]’), these additional sentences contribute to belief in the full statement derived from the first sentence.

In some cases, we know that a TF is active under a given GC, and we find a sentence stating that the target gene is activated under those precise conditions, as well as another sentence saying that the target gene is activated by that TF. Given their consistency, these two sentences complement each other. Consider the following example. FNR is a protein known to be active under anaerobic conditions. A sentence mentions that ‘FNR inhibits the expression of *yfgF*’, and another sentence say that ‘the expression of the gene *yfgF* is inhibited under anaerobic conditions’.

- FNR [−] *yfgF*
- [−] *yfgF* [anaerobiosis]

It was attractive to hypothesize the (FNR [−] *yfgF* [anaerobiosis]) statement, which was eventually found in an article.

We also have to analyze cases of sentences with mutants, in particular, we need to identify how they are described in a sentence. For example, the mutants that we found in this work were described as ΔsoxRS, ΔsoxS and ΔoxyR. Once we are able to identify mutants, we will be able to enrich our consistency when combining information on mutant and TF effects, which should be opposite under the same GCs.

We think it will also help to implement tools that identify the location within the paper (abstract, images, tables, etc.) of pieces of knowledge like RIs and GCs.

## Conclusions and future work

The curation experiment reported here clearly shows that efficient text-mining tools coupled with a customized interface can significantly increase the efficiency and productivity of specific biocuration activities. We were able to identify 100% of the RIs contained in RegulonDB, both for OxyR and for additional TFs described in this corpus of 48 papers by inspecting only 11.4% of the sentences in these papers. Thus, the quality of our traditional curation is preserved in this new process that is based on reading only the set of informative sentences generated by ODIN.

The main goal of this exploratory project is to apply the technology offered by the customized ODIN to learn and sketch a more comprehensive strategy for the semiautomated, or automated if possible, extraction of all GCs present in the full corpus of >5000 papers supporting the mechanisms available in RegulonDB, but that await curation.

Our hypothesis that identification of sentences mentioning target genes and an effect would also identify information on the GCs worked very well, as illustrated by the excellent results obtained. A total of seventy six percent of the OxyR interactions obtained now have identified GC. Additionally, we also identified 67 GCs without identifying the TF, as described in Table 5. As already mentioned, identifying the GCs corresponding to every RI for >200 TFs of the best-characterized regulatory bacterial network will be an important step forward in the overall value of RegulonDB, with an important impact in the understanding of the functioning of this network.

It is important to remember that the corpus of papers we evaluated was generated based on OxyR regulation as curated in RegulonDB, which, given the complex regulation by multiple TFs, also included papers dealing with other TFs. We were able to identify RIs and GCs for several of them. These can be further validated when performing the same curation strategy but in the context of the set of their specific corpus of related papers. This would provides additional opportunities to enhance the reliability of automated extraction strategies within complex biological regulatory networks.

The strategy was not specifically designed for papers already curated and with RIs. This set of previously curated papers was used to test the strategy and measure its efficiency; moreover, the set contains papers that have no data on RIs or GCs. The same strategy will be used with similar sets of papers for the many more TFs and their GCs. We will be able to reestimate its efficiency, which we expect to be similar because there are no reasons for the system to behave differently. Certainly, we are dealing with the same type of knowledge, always about gene regulation, and therefore the same set of verbs for describing activation and repression of target genes are used, except that the names of genes, TFs and GCs will change accordingly for each set of papers. As we proceed, we will learn and improve the system designed precisely for biocuration of microbial gene regulation. Using this technology within precurated literature in a much larger set of papers will enhance the robustness of our measurement of efficiency. This will support the utilization of this tool for the sake of our everyday curation. Furthermore, given the evolutionary conservation of the logics of gene regulation, we believe that this technology can be applied to literature dealing with other organisms, provided their corresponding list of genes is available.

We will gradually automatize much of the most repetitive activities of the curation process, and therefore free up the creative resources of the curators for more challenging tasks, and make possible a much more efficient and comprehensive curation process.
